# Surgical and Patient-Reported Outcomes After Mastectomy and Implant-Based Prepectoral Reconstruction Using TIGR® Synthetic Mesh

**DOI:** 10.7759/cureus.61052

**Published:** 2024-05-25

**Authors:** Shiveta Razdan, Goran A Ahmed, Gayatri Vishwakarma, Chwanrow Baban, Alexandra Tenovici

**Affiliations:** 1 Breast Surgery, Amrita Institute of Medical Science and Research, Faridabad, IND; 2 Breast Surgery, Frimley Health NHS Foundation Trust, Surrey, GBR; 3 Biostatistics, Zydus Research Centre, Ahmedabad, IND; 4 Breast Surgery, University Hospital Limerick, Limerick, IRL

**Keywords:** prepectoral reconstruction, tigr matrix, mibr, breast cancer, quality of life, acellular dermal matrix, synthetic mesh, breast reconstruction

## Abstract

Background

Single-stage direct-to-implant (DTI) breast reconstruction after mastectomy has gained popularity over the last decade, thanks to the wide use of biological matrices and synthetic meshes. Despite their high cost, there is no evidence of superior outcome from the biological matrices compared to the synthetic meshes. In this study, we aimed to evaluate our experience with TIGR, a synthetic, long-term absorbable mesh, in mastectomy and immediate breast reconstruction (MIBR) with a focus on patient-reported outcomes (PROMs).

Methods

This was a single-trust prospective quality improvement study conducted between 2017 and 2019. The main objectives were complication rates including infection, implant loss, and other surgical complications in patients undergoing TIGR mesh-assisted MIBR in the prepectoral plane for either cancer or risk reduction. PROMs were measured using the validated European Organisation for Research and Treatment of Cancer (EORTC) breast questionnaire module. Clinical evaluations were conducted at one week, three weeks, and 12 months postoperatively. All patients provided written consent, and the audit was registered with the Quality Improvement Department of the organization.

Results

One hundred and twelve meshes were used in 93 patients with a mean age of 49 (24-75) years and a body mass index (BMI) of 23.4 (19.1-29.6). During the follow-up period, complications occurred in 26 patients (28%), including infection in four (4.3%), complete skin flap necrosis in one (1%), partial flap necrosis in three (3.2%), and implant loss in four (4.3%) patients. PROM data from 41 individuals indicated a moderate overall quality of life (82.7%), with high functional domain scores with relatively lower emotional functioning scores. Symptom domains generally scored poorly except for body image and sexual functioning.

Conclusion

Mastectomy and immediate prepectoral breast reconstruction using TIGR mesh is safe with low major complication rates. It is associated with high functional and quality of life scores but low scores in symptom domains which could be multifactorial. However, limitations due to study type and follow-up duration suggest caution in generalizing findings.

## Introduction

Breast cancer diagnosis leads to mastectomy in approximately one-third of patients, and the option of breast reconstruction, either immediate or delayed, is considered a crucial quality measure in global breast cancer care [1]. Immediate implant-based breast reconstruction has gained popularity due to its accessibility, cost-effectiveness, and improved aesthetic outcomes due to the preservation of the skin envelope [2]. Traditionally, the implant was placed in the subpectoral plane and was a two-stage procedure [3]. Over the last decade, prepectoral reconstruction has become a popular technique to avoid the animation deformity associated with subpectoral reconstruction. Prepectoral reconstruction may also lead to less incidence of postoperative acute and chronic pain associated with raising the pectoral muscle [4,5].

The use of acellular dermal matrices (ADMs) and synthetic meshes has made single-stage direct-to-implant (DTI) reconstruction a common safe practice [6]. Despite the common use of biological matrices, their limitations, including cost and global availability, have prompted research into low-cost synthetic meshes as potential alternatives. Synthetic meshes are also the first choice for people who would like to avoid animal products. Examples of such synthetic meshes include Vicryl (absorbable), TIGR (long-term absorbable), and TiLOOP (non-absorbable) [7,8]. While ADMs are widely used, there is no strong evidence for their superior outcomes compared to synthetic meshes. A recent systematic review found lower infection and seroma rates in synthetic meshes compared to ADMs but no significant difference in re-operation rates and implant removal [9]. However, there is no strong evidence comparing patient-reported outcomes (PROMs) between synthetic meshes and biologic matrices.

Some studies have shown safety and low complication rates of TIGR mesh [8,10]. However, a comprehensive assessment of PROMs is not widely studied. In our trust, we have been using TIGR mesh for reconstruction for several years. It provides an alternative to patients wishing to avoid animal products and also for patients with religious restrictions against porcine and bovine products. This is a quality improvement initiative, evaluating our surgical outcomes and PROMs with TIGR mesh-assisted prepectoral reconstruction.

## Materials and methods

This was a prospective service evaluation conducted between 2017 and 2019 in two breast units of the same NHS Foundation Trust. Our primary objective was to assess the safety and efficacy of TIGR® mesh (Novus Scientific, Uppsala, Sweden) in patients undergoing immediate breast reconstruction following skin or nipple-sparing mastectomy in the prepectoral plane for either breast cancer treatment or risk reduction. Additionally, we aimed to evaluate PROMs related to the procedure. Consecutive patients who had TIGR mesh for reconstruction based on their choice or surgeon's discretion were included. 

Prior to participation, written informed consent was obtained from all women enrolled in the study. The audit was registered with the Quality Improvement Department of the organization (registration no. CB048). Clinical evaluations were conducted at one week, three weeks, and 12 months postoperatively, with additional assessments as needed. Collected demographic data encompassed factors such as age at surgery, body mass index (BMI), smoking history, comorbidities, details of radiation therapy and chemotherapy, follow-up visits post-surgery, and any encountered complications.

Surgical procedure

In this cohort of patients, the incision choice was made by the operating surgeon along with the patient considering oncological details such as safety of the nipple preservation. Once the mastectomy was performed, the cavity was washed from tissue debris. The pocket of the implant was created around a sizer by anchoring the implant like in a hammock while molding and reducing the excess of mesh. The mesh bearing the implant was anchored to the pectoralis muscle fascia with slow re-absorbable sutures at 11, 12, and 1 o'clock as well as the lower inner quadrant and lower outer quadrant, although there was minor variation among surgeons in anchoring points. The number of anchor points used was kept to a minimum necessary to keep the implant in place. A suction drain was used in all cases to allow drainage of the seroma and facilitate early integration. Local anesthetic was used for postoperative pain or as a top-up of the serratus and/or pectoral block done by the anesthetist. The drains were maintained until a two consecutive day output was low (30-50 ml) and stable. Deep venous thrombosis (DVT) prophylaxis was used as per the trust protocol, and antibiotic practices varied among surgeons from a single dose to 48-hour cover to antibiotic treatment for the length of time the drains were left in. The patients were seen after one week for wound check and then on according to their progress until histology results. The patients were discharged from the surgeon's care following the results clinic at 3-4 weeks postoperative or when the wounds were reported healed and the patient ready for the adjuvant treatment.

PROM scoring method and analyses 

PROM assessments utilized the validated European Organisation for Research and Treatment of Cancer (EORTC) breast questionnaire module, specifically designed for breast reconstructive surgery [11]. The questionnaires were sent out 18-36 months post-surgery. We employed a comprehensive scoring method using well-established tools, including the European Organisation for Research and Treatment of Cancer Quality of Life Questionnaire Core 30 (EORTC QLQ-C30), QLQ-BR23 (Breast Cancer-Specific Module), and BRECON23 (Breast Reconstruction Questionnaire). These instruments cover a wide range of domains, encompassing physical, emotional, social, and functional well-being, providing a holistic perspective on the impact of cancer and its treatment on patients' lives. Scores were calculated by transforming responses into standardized scores, allowing for quantitative analysis. Responses were aggregated and transformed into scores ranging from 0 to 100 using Q score software for each domain. Higher scores indicate greater satisfaction rates. To address missing data, we employed multiple imputations, as recommended in the questionnaire guidelines, using the Statistical Package for Social Sciences (SPSS). In our study, a portion of the dataset contained missing values, necessitating the use of multiple imputations to address these gaps. However, the proportion of missing data that underwent imputation did not exceed 50% of the total dataset. Multiple imputations were conducted with a regression-based approach, leveraging observed associations to predict and replace missing values. This method, executed multiple times to account for uncertainty, facilitated robust and statistically sound analyses, contributing to a nuanced evaluation of the quality of life dimensions in breast cancer patients. 

Statistical analysis

Descriptive statistical methods are used for summarizing and communicating key characteristics of data. For continuous outcomes, measures such as the mean and standard deviation (SD) were used. Additionally, the median and interquartile range (IQR) offer robust alternatives, particularly in the presence of skewed distributions, as they are less sensitive to extreme values. For categorical outcomes, frequencies and percentages were reported elucidating the distribution of different categories within the dataset. IBM SPSS Statistics for Windows, Version 24.0 (Released 2016; IBM Corp., Armonk, New York, United States) was used to analyze the data. 

## Results

The study encompassed 112 reconstructions in 93 consecutive patients with a mean age of 49 years (range: 24-75 years), all of whom underwent prepectoral breast reconstruction utilizing TIGR mesh. A total of 112 meshes were used in our study. None of the patients in our study was an active smoker though three had a history of previous smoking. BMI ranged from 19.1 to 29.6 with a mean of 23.4 kg/m^2^. The primary surgical indication was invasive ductal carcinoma, accounting for 50.8% of cases, followed by risk-reducing mastectomy in high-risk patients, including those with BRCA1/2 mutations, p53 carriers, and Li-Fraumeni syndrome, performed in 24.1% of patients. Other surgical indications comprised ductal carcinoma in situ (13.4%) and invasive lobular carcinoma (9.8%). Among the patients, 19 underwent bilateral procedures. Regarding the choice of implants and expanders, a notable 50.8% of patients received an expander/implant hybrid device, while 25% had expanders alone, and 24.1% had fixed-volume silicone gel implants. Nine patients received neoadjuvant chemotherapy, while adjuvant chemotherapy and radiotherapy were administered to five and 11 patients, respectively. Follow-up durations ranged from 3 to 12 months. Table 1 shows patient and tumor characteristics. 

**Table 1:** Patient and tumor characteristics IDC: invasive ductal carcinoma; DCIS: ductal carcinoma in situ; ILC: invasive lobular carcinoma; ER: estrogen receptor; PR: progesterone receptor; HER-2: human epidermal growth factor receptor; BRCA: breast cancer gene

	Number of patients (N)	Percentage (%)
Laterality		
Unilateral	74	79.56
Bilateral	19	20.43
Mutations		
BRCA 2	4	3.5
BRCA 1	3	2.6
Li-Fraumeni	1	0.89
p53 carrier	1	0.89
	Number of reconstructions (n)	Percentage (%)
Diagnosis		
IDC	57	50.8
Risk-reducing surgery	27	24.1
DCIS	15	13.4
ILC	11	9.8
Adenoid cystic	1	0.9
Apocrine carcinoma	1	0.9
Pathological grade		
1	6	5.3
2	44	39.2
3	20	17.8
Receptor profile		
ER positive	53	47.3
PR positive	48	42.8
HER-2 positive	12	10.7

In our study, a total of 26 patients (28%) of the study population experienced various complications as detailed in Table 2. Infection occurred in four patients, representing 4.3% of the cohort. Complete flap necrosis was observed in one patient (1%), while partial skin necrosis occurred in three patients (3.2%). Capsular contracture was seen in one patient (1%), implant loss was seen in four patients (4.3%), and exchange of the implant was conducted in three patients (3.2%). Seroma formation was observed in two patients, accounting for 2.1% of the study population. The incidence of some of the complications was compared to international joint consensus on prepectoral breast reconstruction [12].

**Table 2:** Surgical parameters and postoperative complications Reference: [12]

Complications	Number of patients (N)	Percentage (%)	Desirable standard*
Infection	4	4.3	<10%
Implant loss	4	4.3	<10%
Change of implant revision	3	3.2	<10%
Partial necrosis	3	3.2	NA
Nipple excision	2	2.1	NA
Complete flap necrosis	1	1	NA
Capsular contracture	1	1	NA
Type of breast prosthesis used			
Expander-implant hybrid device	57	50.8	
Expander	28	25	
Fixed-volume silicone gel implants	27	24.1	

**Figure 1:**
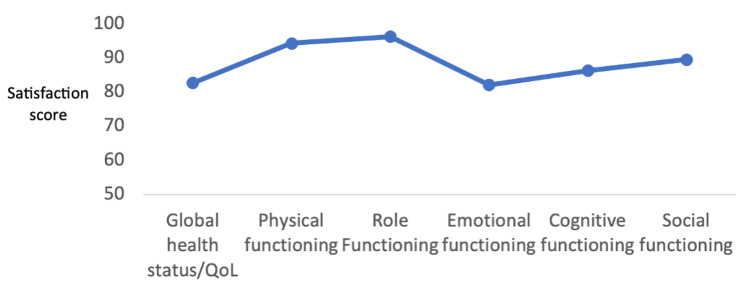
Graphic representation of quality of life scores

PROM data were collected from 41 individuals out of 93 included subjects, providing insights into their perceived quality of life. The questionnaires were sent out 18-36 months after surgery. The overall quality of life score averaged at 82.7%, indicating a moderate level of well-being within the sampled population (Table 3). Within the functional domain, patients reported high levels of role functioning, with a maximum score of 96.3%. However, emotional functioning received a comparatively lower score of 82.1%, suggesting potential challenges in this aspect of well-being (Figure 1). In exploring other symptom domains, generally lower scores were observed, except for body image, future perspective, and sexual functioning. Notably, these domains showed higher scores, and this positive trend was attributed to the impact of adjuvant treatments administered post-surgery (Table 3).

**Table 3:** QOL scores *Median satisfaction of 11.1: This suggests that the middle value of your satisfaction data is 11.1. In a typical satisfaction survey or scale, this could represent a moderate level of satisfaction. **IQR of 30.6: The IQR represents the spread of the central 50% of the satisfaction data. A larger IQR indicates greater variability in satisfaction scores within the middle 50% of respondents. For example, if the IQR is 30.6 on a scale of 1-100, it suggests that the satisfaction scores for a significant portion of respondents vary by around 30 points. QOL: quality of life; IQR: interquartile range

QOL scale	Mean	SD	Median*	IQR**	Interpretation of QOL
Global health status/QOL	82.7	18.3	83.3	22.9	Good
Functional scale					
Physical functioning	94.3	7.2	93.2	6.7	Good
Role functioning	96.3	8.8	100.0	0.0	Good
Emotional functioning	82.1	21.8	83.3	33.3	Good
Cognitive functioning	86.3	19.9	100.0	33.3	Good
Social functioning	89.6	20.3	100.0	29.2	Good
Symptom scales/items					
Fatigue	19.2	19.9	11.1	30.6	Poor
Nausea and vomiting	2.5	7.1	0.0	0.0	Poor
Pain	11.3	19.0	0.0	16.7	Poor
Dyspnea	4.2	13.5	0.0	0.0	Poor
Insomnia	25.0	28.0	33.3	33.3	Poor
Appetite loss	8.3	18.1	0.0	0.0	Poor
Constipation	14.2	23.7	0.0	33.3	Poor
Diarrhea	6.7	13.5	0.0	0.0	Poor
Financial difficulties	12.5	23.5	0.0	33.3	Poor
Symptoms					
Systemic therapy	13.1	12.0	9.5	14.3	Poor
Hair loss	33.3	21.4	11.1	0.0	Poor
Arm symptoms	13.9	16.6	11.1	30.6	Poor
Breast symptom	12.1	14.0	8.3	16.7	Poor
Body image	72.1	26.8	79.2	43.8	Good
Future perspective	54.2	26.9	66.7	33.3	Good
Sexual functioning	80.0	25.9	83.3	33.3	Good
Sexual enjoyment	4.7	20.9	2.0	2.8	Poor
Treatment side effects	11.7	17.4	0.0	16.7	Poor
Donor site symptom	30.6	8.9	33.3	0.0	Poor
Loss of nipple	30.8	13.9	33.0	0.0	Poor

**Figure 2:**
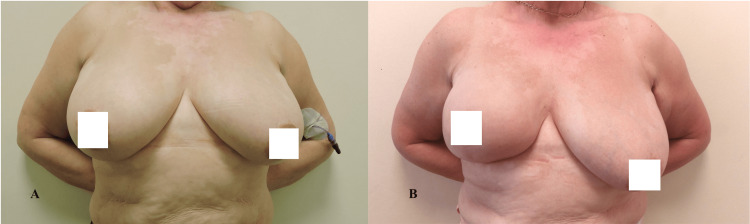
(A) Preoperative photos of a patient with right breast cancer after NACT. (B) Postoperative photo after right skin-reducing and nipple-sparing mastectomy using implant/expander hybrid device and TIGR mesh NACT: neoadjuvant chemotherapy

**Figure 3:**
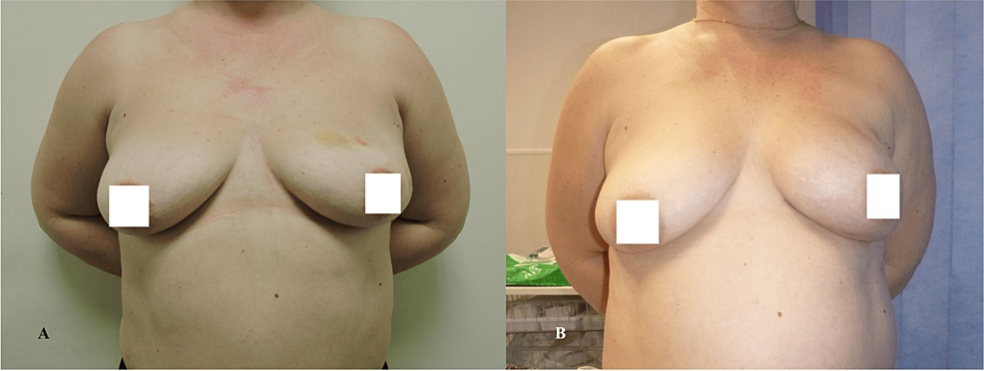
(A) Preoperative photos of a patient with left breast cancer. (B) Postoperative photo after left nipple-sparing mastectomy with direct-to-implant-based reconstruction using TIGR mesh

**Figure 4:**
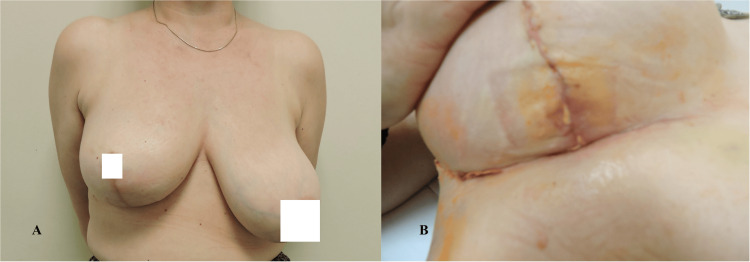
(A) Postoperative patient with right skin-reducing mastectomy and implant-based reconstruction using TIGR mesh. (B) Same patient with partial skin necrosis at the suture line managed conservatively

The presented descriptive statistics encapsulate a comprehensive profile of various health-related parameters assessed in a sample of 40 individuals (Table 4). The measures encompass physical functioning (PF), role functioning (RF), emotional functioning (EF), cognitive functioning (CF), social functioning (SF), and global health status. The means and variability of these factors are elucidated, offering insights into the diverse aspects of individuals' well-being post-medical interventions. Notably, physical and role functioning exhibited high mean scores, reflecting robust health in these domains. Conversely, emotional functioning displayed a broader range, indicative of varied emotional states within the cohort. The data also encompass specific symptoms and side effects experienced, such as fatigue (FA), pain (PA), insomnia (SL), and sexual functioning. Notably, body image and future perspective demonstrated noteworthy mean scores, suggesting potential areas of impact and concern. Figures 2-4 show the preoperative and postoperative photographs of some of the patients.

**Table 4:** Descriptive statistics of various health parameters in the given number of patients

Measured parameters	N	Minimum	Maximum	Mean	Std. deviation
PF (physical functioning)	40	73.33	100.00	94.33	7.17
RF (role functioning)	40	66.67	100.00	96.25	8.84
EF (emotional functioning)	40	8.33	133.33	82.08	21.81
CF (cognitive functioning)	40	33.33	133.33	86.25	19.93
SF (social functioning)	40	33.33	133.33	89.58	20.22
Global health status	40	25.00	100.00	82.71	18.33
FA (fatigue)	40	0.00	77.78	19.17	19.97
NV (nausea and vomiting)	40	0.00	33.33	2.50	7.11
PA (pain)	40	0.00	100.00	11.25	19.02
DY (dyspnea)	40	0.00	66.67	4.17	13.48
SL (insomnia)	40	0.00	100.00	25.00	27.99
Appetite loss	40	0.00	66.67	8.33	18.10
Constipation	40	0.00	100.00	14.17	23.74
Diarrhea	40	0.00	33.33	6.67	13.50
Financial difficulties	40	0.00	100.00	12.50	23.49
Systemic therapy	40	0.00	47.62	13.10	12.04
Hair loss	40	0.00	100.00	33.33	21.35
Arm symptoms	40	0.00	66.67	13.89	16.64
Breast symptom	40	0.00	75.00	12.08	13.99
Body image	40	0.00	100.00	72.08	26.79
Future perspective	40	0.00	100.00	54.17	26.89
Sexual functioning	40	0.00	133.33	80.00	25.93
Sexual enjoyment	40	0.00	133.33	4.71	20.90
Treatment side effects	40	0.00	66.67	11.67	17.38
Donor site symptom	40	0.00	55.56	30.56	8.98
Loss of nipple	40	0.00	66.67	30.83	13.89

## Discussion

This was an audit and evaluation of outcomes of the use of TIGR mesh in mastectomy and immediate breast reconstruction (MIBR) in the prepectoral plane in a large cohort of patients. Our findings show overall complication rates and implant loss were comparatively low when compared with findings from other studies in the literature using synthetic meshes and ADMs [13,14]. Furthermore, the satisfaction levels in PROMs with prepectoral reconstruction in our study were generally favorable similar to the outcomes documented in other studies [14,15]. These findings suggest that, on the whole, patients undergoing immediate prepectoral breast reconstruction with synthetic mesh in our cohort expressed a satisfactory experience and perceived benefits from the intervention without an increase in complications. 

The use of meshes and ADMs in breast surgery has enabled single-stage, direct-to-implant breast reconstruction in prepectoral or dual planes with no or mild muscle morbidity. These meshes serve as a supportive scaffold, fostering tissue ingrowth and providing internal support to the implant, thereby contributing to the maintenance of natural breast ptosis and the inframammary fold. While biological meshes offer advantages, such as promoting tissue integration and internal support and better mammary fold definition, they are not without complications. Issues like infection, cellulitis, seroma, skin flap necrosis, wound dehiscence, implant loss, and the high cost of the mesh are potential drawbacks [16]. On the other hand, synthetic meshes, an alternative to biological meshes, have been employed to address similar issues but are less widely adopted globally. The utilization of synthetic meshes introduces its own set of considerations, and the choice between biological and synthetic options may depend on various factors including patient-specific characteristics and surgeon preferences. In a large recent systematic review comparing synthetic meshes and ADM, outcomes and complications profile, as well as quality of life and aesthetic outcomes, were equivalent in synthetic mesh compared to ADM in MIBR [17]. However, the literature is lacking in high-quality evidence, and prospective studies are needed with long-term follow-ups, to compare the effectiveness and potential complications associated with the use of biological and synthetic meshes in implant-based breast reconstruction. 

TIGR is an innovative long-term resorbable synthetic mesh characterized by two distinct resorbable fibers that degrade at different rates. This unique feature enables the mesh to seamlessly integrate with the body tissues in a controlled manner, facilitating a stable transfer of the implant's weight from the matrix to the patient's tissues, as elucidated in a pre-clinical study by Hjort et al. [18]. Notably, TIGR offers robust support in the initial six months, crucial for the early stages of healing, and undergoes complete resorption over three years.

A comprehensive exploration of the application of TIGR mesh is presented in the extensive series by Becker and Lind, constituting the largest body of work on its use to date [8]. The versatility of TIGR mesh is evident in its application across reconstructive, revision, and aesthetic surgical procedures. This series not only highlights the mesh's capacity to address various surgical needs but also underscores its potential to enhance outcomes across a spectrum of surgical interventions. The controlled degradation and resorption timeline of TIGR contributes to its appeal, presenting a promising option in the realm of synthetic meshes for a diverse range of surgical applications.

Studies have shown a higher infection rate with the use of ADM compared to synthetic mesh or no mesh after MIBR. A recent systematic review found a 2.97 times higher infection rate with ADM compared to no mesh but no significant difference between synthetic meshes and no mesh use in infection rate [9]. In another literature review by Ellis et al., infection rates with ADM in the literature ranged between 0.2% and 35.8%, while with synthetic mesh use in breast reconstruction, studies report an infection rate between 1.3% and 6.1% [13]. Notably, the infection rate reported in our series stands at 4.3%. The presence of *Staphylococcus aureus* forming a biofilm with ADM is postulated as a potential explanation for the higher infection rates observed in biological matrices [19]. Another reason for increased infection rate after ADM use is higher seroma rates. In the context of our study, the observed seroma rate was 2.1%. The incidence of seroma formation varies across different matrices and meshes. For ADMs, reported rates range from 1.5% to 24.3%, while synthetic meshes exhibit a seroma formation rate in the range of 0-5.7% [13].

The differing propensities for seroma formation between ADM and synthetic meshes can be attributed to the specific characteristics of these materials. ADMs, with their smooth surface, may foster increased fluctuation between the matrix and subcutaneous tissue, potentially leading to a higher incidence of seroma formation. On the other hand, meshes, characterized by a rough surface, facilitate quicker interaction with subcutaneous tissue, resulting in less seroma formation [20]. 

In our study, the incidence of complete flap necrosis was observed in 1% of patients, while partial flap necrosis occurred in 3.2%. When compared to the literature, reported rates of skin flap necrosis for meshes range from 1.8% to 4.3%, and for matrices, the range is wider, spanning from 1.4% to 24.3% [13]. Several factors contribute to these varying rates, including patient comorbidities, smoking status, thin mastectomy flaps, overexpansion, and specific surgical techniques. In a small prospective trial, the use of an inverted T-shaped incision was found to be associated with a particularly elevated skin necrosis rate of 30% [21]. These findings highlight the multifactorial nature of flap necrosis, emphasizing the importance of considering patient-specific factors and surgical approaches to ensure flap viability. 

In our study, the rate of implant loss was recorded at 4.3% at 12 months. When comparing this to the existing literature, the reported implant loss rates for both meshes and matrices range from 0% to 8.7%. Notably, some studies suggest a higher incidence of implant loss with the use of ADMs, potentially due to increased seroma formation [13,16].

Differentiating capsular contracture rates between matrices and meshes lacks conclusive evidence from high-quality studies. Reported rates range from 1.3% to 8.6% in meshes and from 0.4% to 8.1% in matrices, indicating a generally comparable occurrence between these materials [13]. In our study, we observed a low rate CC of 1% at 12 months which is a relatively short period for this specific outcome. 

In this cohort group, we conducted a comprehensive analysis of the quality of life and patient satisfaction following immediate breast reconstruction utilizing synthetic mesh. Different studies in the literature have compared the use of biological and synthetic mesh in breast reconstruction for quality of life and found not much difference between the uses of both kinds of meshes [22]. Our study revealed an overall quality of life score of 82.7%, indicating a moderate level of satisfaction among patients with the treatments provided. As compared to other studies, physical functioning in our study was 94.3% compared to the study done by Negenborn et al. where the physical functioning score was only 80.5% [23]. Conversely, suboptimal scores in the symptom domain were observed. However, it is important to contextualize these findings by considering that a significant proportion of the study participants underwent systemic chemotherapy and radiotherapy which are known to negatively impact PROM scores [24]. These therapeutic interventions, while contributing to lower scores in symptom domains, did not necessarily compromise patients' perceptions of body image, as evidenced by a satisfactory rating of 72.1%.

These insights shed light on the nuanced impact of immediate reconstruction with synthetic mesh on patients' quality of life and satisfaction. The consideration of PROMs, along with the acknowledgment of the influence of adjuvant treatments on specific domains, contributes to a comprehensive understanding of the overall treatment experience.

This quality improvement initiative has several limitations. Firstly, due to the nature of the work, there is no randomization and no control group which risks selection bias. This in turn limits the ability to draw causal conclusions or establish the efficacy of specific interventions. Secondly, the absence of baseline Breast Q scoring data represents a notable limitation. Having baseline data would have enabled a more robust comparison of the quality of life before and after reconstruction. This gap in data prevents the examination of changes in the general health status of patients over time, and it precludes the performance of tests for response shift, which could have offered additional insights into the dynamics of PROMs. Furthermore, the relatively short follow-up period, especially concerning complications such as capsular contracture, is acknowledged as a limitation. A longer-term follow-up would have allowed for a more comprehensive assessment of the incidence and persistence of complications over an extended duration, providing a better understanding of the durability of the outcomes.

## Conclusions

The use of TIGR mesh in immediate prepectoral breast reconstruction seems to be associated with relatively low complication rates including infection and implant loss. Additionally, our cohort had an overall moderate level of quality of life score and a good level of functioning but relatively low scores in symptom domains. However, due to the nature of the study and relatively short-term follow-up, caution must be exercised in generalizing findings and highlighting areas for improvement in future research endeavors.
